# Mitochondria break through cellular boundaries

**DOI:** 10.18632/aging.102103

**Published:** 2019-07-13

**Authors:** Jiri Neuzil, Michael V. Berridge

**Affiliations:** 1 School of Medical Science, Griffith University, Southport, Qld 4222, Australia; 2 Institute of Biotechnology, Czech Academy of Sciences, Prague-West 252 50, Czech Republic; 3 Malaghan Institute of Medical Research, Wellington 7060, New Zealand

**Keywords:** mitochondrial transfer, mitochondrial DNA, respiration, *de novo* pyrimidine synthesis, cancer

In our recent research, we have used respiration-deficient tumour cells to challenge the dogma that mitochondria with their genome are constrained within cells in the body, and to question the concept that mitochondria are primarily the powerhouse of the cell.

Our results have shown that mitochondria move from normal cells in the body to tumour cells without mitochondrial DNA (mtDNA), resulting in respiration recovery and the ability to grow as tumours [1, 2]. Almost a decade earlier, a similar phenomenon had been show in co-cultures of human tumour cells lacking mtDNA with mesenchymal stem cells [3]. The strength of both of these ground-breaking studies was that they used mtDNA polymorphisms to show repopulation of tumour cells with mtDNA, establishing the origin of mitochondria in the donor cells.

Using the same metastatic melanoma and breast carcinoma cell lines utilised to demonstrate intercellular mitochondrial transfer *in vivo*, we applied genetic approaches to determine whether the inherent failure of tumour cells devoid of mtDNA to form tumours was due to their inability to generate mitochondrial ATP or due to a failure of respiration, which together comprise OXPHOS [4]. Rather unexpectedly, tumour cells without the nuclear *Atp5b* gene that encodes a subunit of ATP synthase required for ATP synthesis, were able to form tumours by using glycolytic ATP. In contrast, cells without *Dhodh*, a nuclear gene that encodes dihydroorotate dehydrogenase (DHODH), a rate-limiting enzyme in *de novo* pyrimidine biosynthesis and nucleic acid production that depends on coenzyme Q (CoQ) oxidised via the respiratory activity of mitochondria, did not result in tumour formation. In addition, expression of alternative oxidase (AOX) in cells without mtDNA provided sufficient respiration and redox-cycling of CoQ to fuel pyrimidine synthesis and, consequently, tumour growth. Together, these results explain why cell lines that lack mtDNA are auxotrophic for uridine and need to be supplied with this substrate Further, cell lines derived from tumours that grew from *Atp5b* gene knockout cells were glycolytic, indicating that tumour formation by these cells relies on DHODH-dependent respiration and on ATP generated by glycolysis.

The discovery that tumour cells do not need mitochondrial ATP to form tumours was unexpected and is novel. While the view that many tumours are often highly glycolytic even in the presence of oxygen (the Warburg effect) is now widely accepted almost a hundred years after it was first proposed, Warburg’s view was that “cancer cells can obtain approximately the same amount of energy from fermentation as from respiration” [5]. More recent estimates indicate that cancer cell lines can derive up to 70-80% of their ATP from glycolysis, and our results revealed that the breast cancer and melanoma cells used in our studies derive 50-60% of their ATP from OXPHOS [4].

How do these results relate to normal physiology and aging, and to diseases other that cancer such as neurodegenerative and neuromuscular pathologies? First, the lack of robust tools to track mitochondrial movement between cells in the body makes it difficult to determine whether intercellular mitochondrial transfer is a common occurrence or a relatively rare event (or whether it does occur at all). The problem is compounded by heteroplasmy of mtDNA, that is, the presence of more than one mitochondrial genotype and varying levels of different genotypes in different cells. Inbred mice are ideal models for mitochondrial transfer studies because they are homoplastic, and the tumours we have used have mtDNA polymorphisms that distinguish between the tumour cells and the mouse from which they were derived. Bone marrow transplantation studies in our laboratory have not identified detectable mitochondrial transfer between donor and recipient hemopoietic cells in mice. Mitochondrial uptake by damaged cells has also been described, both for free extracellular mitochondria [6] and for mitochondria in micro-vesicles [7], and therapeutic applications of mitochondrial therapy following cardiac damage, brain ischemia perfusion injury and lung inflammation have been described suggesting physiological mechanisms of mitochondrial movement between cells [8]. Regarding aging, it is too early to make predictions about the role and potential therapeutic use of mitochondrial transfer and therapeutic transplantation to promote healthy aging, yet, given our recent results and the work by others, we expect this field to become a little more crowded in the years ahead.

## References

[1] Tan AS, et al. Cell Metab. 2015; 21:81–94. 10.1016/j.cmet.2014.12.003 25565207

[2] Dong LF, et al. eLife. 2017; 6. 10.7554/eLife.22187 28195532 PMC5367896

[3] Spees JL, et al. Proc Natl Acad Sci USA. 2006; 103:1283–88. 10.1073/pnas.0510511103 16432190 PMC1345715

[4] Bajzikova M, et al. Cell Metab. 2019; 29:399–416. 10.1016/j.cmet.2018.10.014 30449682 PMC7484595

[5] Warburg O. Science. 1956; 123:309–14. 10.1126/science.123.3191.309 13298683

[6] McCully JD, et al. Mitochondrion. 2017; 34:127–34. 10.1016/j.mito.2017.03.004 28342934

[7] Hayakawa K, et al. Nature. 2016; 535:551–55. 10.1038/nature18928 27466127 PMC4968589

[8] Berridge MV, Neuzil J. Clin Exp Pharmacol Physiol. 2017 (Suppl 1); 44:15–20. 10.1111/1440-1681.12764 28409855

